# Effects of anthropogenic activities on microplastics in deposit-feeders (Diptera: Chironomidae) in an urban river of Taiwan

**DOI:** 10.1038/s41598-020-79881-z

**Published:** 2021-01-11

**Authors:** Chun-Ting Lin, Ming-Chih Chiu, Mei-Hwa Kuo

**Affiliations:** 1 Department of Entomology, National Chung Hsing University, Taichung, Taiwan; 2 Institute of Hydrobiology, Chinese Academy of Sciences, Wuhan, China

**Keywords:** Ecology, Ecology, Environmental sciences, Hydrology

## Abstract

The presence of microplastics (MPs) in the environment has generated global concerns. However, the explicit assessment of the effect of multiple anthropogenic activities on the existence of MPs in the freshwater system is scarcely reported. This study quantified anthropogenic activities and analyzed their relationship with MPs on a freshwater organism: the midge larvae (Diptera: Chironomidae). The study took place in an urban river and consisted of comparing the abundance and types of MPs. Our results highlight that, while industrial area was the most important variable contributing to the total MP concentration in midge larvae, residential area also influenced the concentration of microfibers in midge larvae. The impact of a residential area on the relative abundance of microfibers in each sample site was diluted by the proximity to an industrial area. In conclusion, we suggest that industrial areas are a potential source of MP pollution in river sediment, and midge larvae can be a good indicator of the MP concentrations in urban river systems. Quantifying anthropogenic activities can help discern their effects on MP concentration in a river system and promote management strategies.

## Introduction

The impact of microplastics (MPs), < 5 mm in size defined by Arthur et al. (2009), has no longer been neglected in both terrestrial and aquatic environments^1–3^. Cox et al. (2019) estimated that in the American diet, the number of MP particles each person consumed and inhaled annually could be as many as 120,000, which could be underestimated as it excludes bottled water. Therefore, it is not surprising that MPs are already accumulating in the environment, including beaches and estuaries^5,6^, the ocean’s surface^7^, deep marine sediment^8^, the Arctic^9^, the atmosphere^4,10^, and rain drops^11^. Indeed, physical injuries and absorption of environmental contaminants on the MP surface could be involved with ingestion of MPs by organisms^12^, but these biotic influences are seldom studied.

Anthropogenic activities have been well known to influence the existence of MPs in environments^13,14^, but the quantification of these activities is still limited. In the proximity of anthropogenic activities, areas were considered sites with elevated MP abundance in previous research^12–17^. For example, a study in central China showed that the MP concentration of inland surface water was negatively correlated with the distance from an urban area, suggesting the possibility that anthropogenic factors determined how MPs varied in the space^18^. By contrast, a study in Germany indicated that no correlations were found between population density, industrial activities, location of a wastewater treatment plant and MP concentration in river sediment^19^. Nel et al. (2016) also found no relationships between local municipal density and MP density in the water column and sediment of the southern Africa coastline. Nevertheless, both of these studies observed elevated MP abundance in sample sites that were close to anthropogenic activities. Although this fact is reasonable as these populated areas are usually involved with various activities that produce plastics^20^, only Yonkos et al. (2014) have quantified and compared different anthropogenic activities with MP concentration in environments. Many of the existing studies on anthropogenic activities are devoted to human density^14,17^, resulting in ambiguous conclusions that governments could not build effective policy from.

Compared to the marine environment, scientific studies of MPs in freshwater systems is lacking^2^, which is crucial as urban rivers are usually surrounded by densely populated humans, and are considered to be the transporters of MPs to sea^21^. One problem is the comparative lack of indicator species in freshwater systems which can reflect MP pollution. In coastal regions globally, multiple mussel species were studied and proposed as bioindicators^22^, and multiple marine indicator species have already been established^23^. However, potential bioindicators in the freshwater systems are scarcely reported^17,24,25^. Since freshwater species have different ecological niches, it is necessary to identify potential bioindicators that can be used to monitor the microenvironment in freshwater systems. Chironomidae larvae are one of the dominant species with non-selective feeding on sediment in urban rivers^26,27^. Chironomidae are known to consume MPs and to reflect the degree of MP pollution in river sediment^17,28,29^ and therefore were great vectors to evaluate MP pollution in river sediment. In fact, Windsor et al. (2019), who investigated MP concentration in Ephemeroptera and Trichoptera, suggested that organisms inhabiting the sediment or subsurface are more likely to ingest aggregated MPs than those in the water column. For example, smaller MPs, which showed strong aggregation in the environment, were observed to accumulate in the gut of *Chironomus tepperi*, inhibiting food ingestion^31^. This allows us to detect MPs in Chironomidae larvae. Therefore, since the ecological niche of organisms is an important factor influencing the possibility of being a bioindicator of MPs, as deposit-feeders, Chironomidae larvae are believed to be a potential bioindicator, but further analysis is needed.

To the best of our knowledge, this study is the first to analyze the effects of multiple quantified anthropogenic activities on MP presence in organisms in a river system. Specifically, we carried out the investigation of MPs in Chironomidae larvae in the urban river basin of central Taiwan. In addition, Chironomidae larvae could be great bioindicators of MP pollution in urban river systems as they can be dominant in such polluted environments. We investigated two hypotheses: (1) both industrial and residential areas are main sources of MPs in populated areas, resulting in similar contributions of MPs in Chironomidae larvae, and (2) residential areas mainly produce microfibers into urban river sediment. Our study intends to offer constructive advice when devising manageable strategies to mitigate MP pollution in urban river systems.

## Materials and methods

### Study sites and sampling

**Figure 1:**
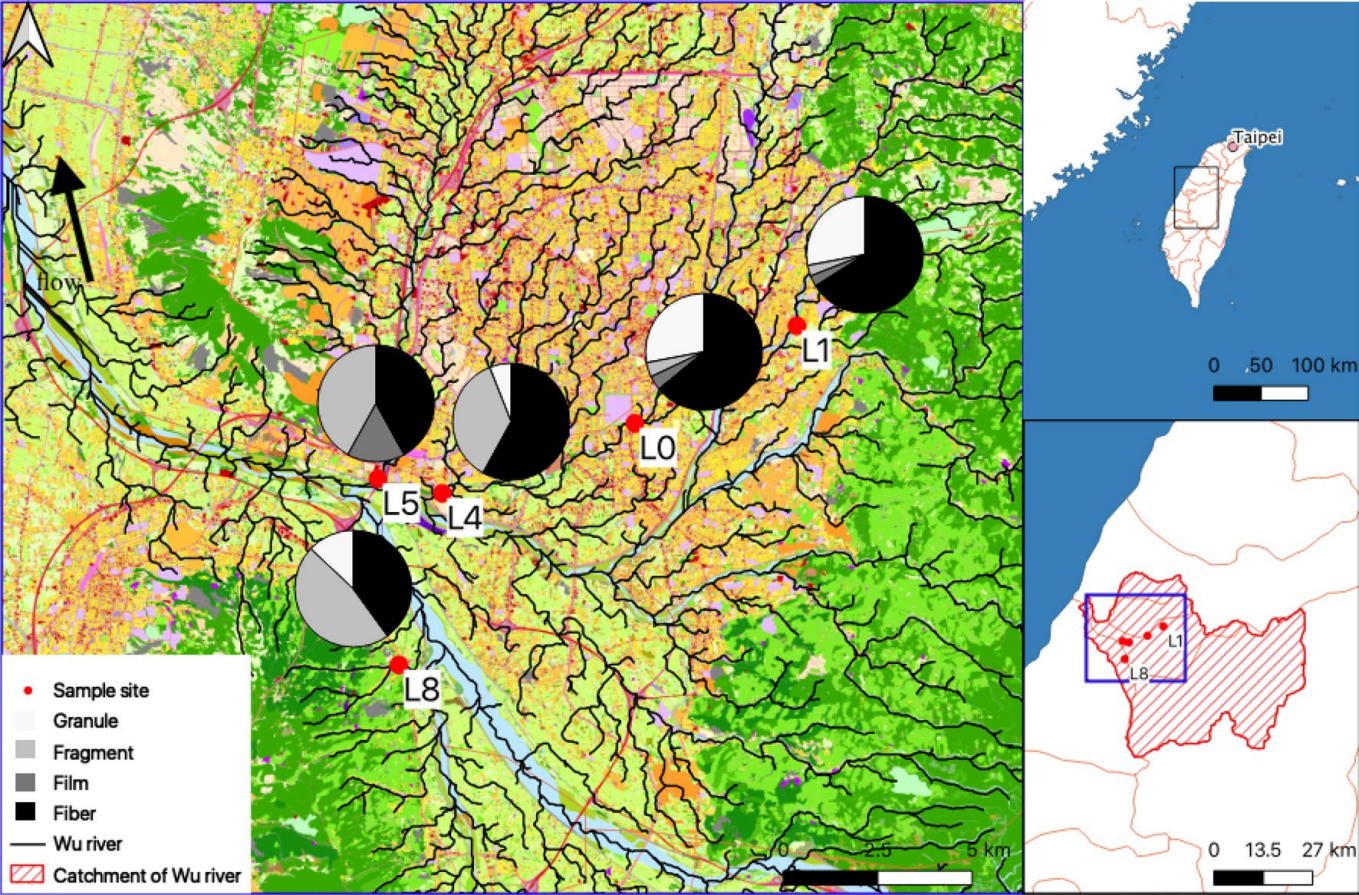
Location of each sample site in the Wu river basin. The hydrographic map was calculated using the module, Terrain Analysis—Channels^65^, by configuring the System for Automated Geoscientific Analyses^66^ (SAGA GIS) in Quantum GIS (QGIS) 3.10, where the pie chart indicates the proportion of four different morphotypes of microplastics at each sample. Different colors in land use raster presented at the background of the main map indicate different land usages. Coordination system: TWD97/TM 2 Zone121 EPSG:3826; Unit: meters.

Between May and June 2019, 5 study sites in the Wu river basin (2025 km^2^, 119 km long) in central Taiwan were visited (Fig. 1). Wu river flows across Taichung city that is the second largest metropolitan area and second most populated area (10% of total Taiwanese population, 2016) in Taiwan^32^. Precipitation in Taichung ranged from 0–768 mm monthly in 2019^33^, indicating the heterogeneity of river flow due to seasonal difference. To avoid the dry period of the river, we collected all samples in the summer (rainy season), on days when it was not raining. One sample was taken at site L0 and three samples were collected at the remaining sites.

The catchment of site L5 includes one of the biggest industrial parks in central Taiwan, while sites L4 and L0 are in a densely populated, residential area. By contrast, the catchments of sites L1 and L8 are not as urbanized as the other sample sites. Overall, site L5 has the largest catchment, industrial and residential areas, and also the highest proportion of industrial area, while site L0 has the largest proportion of residential area. Besides, site L0 has the highest density of residential area in the catchment. Sites L1 and L8 have a low density of anthropogenic activities in the catchment. Sites L4 and L5 have a high density of anthropogenic activities in the catchment (Table 1). There are 3 wastewater treatment plants (WWTPs) in the catchment of site L5, and 1 WWTP in the catchment of site L4. Coordination of sample sites and characterizations of land use in the catchments of the sample sites are listed in Table 1. All sampling was implemented on a stone-based riverbed with a Surber sampler (12 × 12 inch, mesh size: 250 μm) where all organisms on stones in the sample site were washed, and organisms were preserved with 75% ethanol.

**Table 1:** Coordination, catchment, and anthropogenic areas of sample sites.

Sample sites	Coordination (decimal degrees)		Area (km^2^)			Density (%)		Ratio
	Latitude	Longitude	Catchment area	Industrial area	Residential area	Proportion of industrial area	Proportion of residential area	
L0	24.117	120.680	8.69	0.17	1.61	1.94	18.55	9.55
L1	24.141	120.722	43.51	0.21	1.61	0.47	3.70	7.80
L4	24.100	120.630	50.10	1.53	6.46	3.06	12.89	4.21
L5	24.104	120.613	151.33	13.86	10.51	9.16	6.94	0.76
L8	24.059	120.619	4.70	0.04	0.13	0.86	2.77	3.22

Ratio: the ratio of residential area to industrial area.

### Measurement of length and dry weight

In order to mitigate contamination, the liquid used to clean equipment prior to use was filtered. All larvae were washed (especially visible fibers attached on body) and photographed when each body length was recorded by computer software. Three different genera (*Thienemannimyia* spp., *Chironomus* spp., and *Orthocladius* spp.) were detected in this area using a scanning electron microscope (SEM, Figs. S1 and S2) with a taxonomy key^34^, although not all larvae in our samples were identified further. Vacuum filtration was conducted to briefly dry samples before we placed the filter papers (carefully covered by aluminum foil) inside a drying oven (approximately 42 °C, SM 400, Memmert) for at least 24 h. We measured the weights of the filter papers with samples before and after drying using an analytical balance (GR-202 42 g/210 g, A&D company) to calculate dry weights. The dry samples were then placed inside a 150 ml bottle for further analysis.

### Extraction and observation of microplastics

At least 30 ml of 30% hydrogen peroxide was added to the bottle with the dry sample (ensuring that the liquid covered all larvae). Thereafter, samples were heated to 60 °C and 100 °C to breakdown the tissue, following the modification of methodology by Claessens et al. (2013), on a magnetic stirrer (420D, Corning, Mexico). The remaining liquid was vacuum filtered onto 5 μm white qualitative filter paper (No.2, 55 mm, Advantec). Filter papers were preserved in clean petri dishes for subsequent processes. Control settings were also conducted under the same procedure to evaluate any contamination during the experiment.

The filter papers were observed under a Leica M205 C Stereo microscope. We applied visual assessments to identify and enumerate suspected microplastic particles. Representative particles were selected and further observed and photographed using scanning electron microscope (S-3400N). While particles with color were recorded as microplastics, transparent and white particles were considered to be of biological origin and excluded^35^. We applied microplastic concentration (number of microplastics/microgram of sample) for demonstrating the degree of MP pollution. In line with previous studies^36,37^, we categorized microplastics into 4 morphotypes: microgranule, microfilm, microfragment, and microfiber (Fig. S3).

### Quantification of anthropogenic activities

Anthropogenic activities in this study were defined as activities taking place in the industrial area and residential area. Quantification of these areas was induced by a free geographic information system, Quantum GIS 3.10, which was used to calculate the catchment areas of study sites. The Web Map Service (WMS) of land-use map in the Taiwan Map Store^38^ from the National Land Surveying Mapping Center, Ministry of the Interior, Taiwan, demonstrated the distribution of industrial and residential areas. We extracted the representative colors of industrial and residential areas on a map, to receive filtered images with only pixels of industrial or residential areas. As a result, we were able to calculate the proportion of industrial and residential areas in the catchment areas. By mapping the catchment area of the study sites and the proportion of industrial and residential areas, we were able to determine the actual area.

### Data analysis

The difference of body length between sample sites and the difference between the concentration of microplastics was determined by one-way analysis of variance (ANOVA) or Kruskal–Wallis test. Which test was used depended on the results of the Shapiro-Wiki test (normality) and Levene’s test (equality of variances). Site L0 was excluded from statistical analyses in Table S1 since we only collected 1 sample in site L0. A significance level of 0.05 was chosen in each test.

We used a generalized linear mixed model and model selection to explore the effects of catchment and anthropogenic activities on microplastic concentration of Chironomidae larvae. According to certain variables (PIA: proportion of industrial area, IA: industrial area, PRA: proportion of residential area, RA: residential area, CA: catchment area, RA/IA: ratio of residential area to industrial area), we performed 30 generalized linear mixed models with random effects (sample site and sample date). This was to assess the relationship between the MP concentration and environmental variables, which are listed in Table 2 if model delta < 4 (others were listed in Table S3). Furthermore, model selection was conducted based on Akaike information criterion^39^. Corrected Akaike information criterion (AICc) for a small sample size was used^40^. The model with the smallest AICc value is considered to be the best model to describe our field data as we expect it has the smallest loss of information.

**Table 2:** Generalized linear mixed model (delta < 4): relationship between variables and microplastic concentrations (random effects: sample sites, sample dates).

Fixed effects	df	logLik	AICc	Delta	Weight	Conditional R^2^	Marginal R^2^	beta	*p* value	Variables	Sum of weight
PIA	5	− 10.35	39.3	0.00	0.342	0.5882	0.4681	18.8689	0.019	PIA	0.32
logIA	5	− 10.46	39.5	0.23	0.305	NA	0.5362	0.2897	0.022	logIA	0.35
logRA	5	− 11.11	40.8	1.53	0.159	NA	0.4383	0.3186	0.126	logRA	0.15
logCA	5	− 11.60	41.8	2.50	0.098	0.4998	0.1784	0.2813	0.334	logCA	0.16
PRA	5	− 11.67	41.9	2.63	0.092	0.4603	0.1071	5.6596	0.378	PRA	0.09
logIA + logCA	6	− 8.17	42.3	3.06	0.064	NA	0.6422	logIA: 0.7657	< 0.001		
								logCA: − 0.8554	0.004		

*PIA* proportion of industrial area, *logIA* logarithm of industrial area, *logRA* logarithm of residential area, *logCA* logarithm of catchment area, *PRA* proportion of residential area, *df* degree of freedom, *logLik* log likelihood, *AICc* correction of AIC with small sample size, *Conditional r*^*2*^ r-squared explained by both fixed factor(s) and random factors, *Marginal r*^*2*^ r-squared explained by the fixed factor(s) alone. Sum of weights column indicates the relative importance of each variable.

We performed all analyses under R 3.5.1^41^. We analyzed generalized linear mixed models by glmmTMB^42^ and GLMMadaptive^43^, model selection by MuMIn^44^, and R^2^ by performance^45^.

## Results

### Microplastic concentration

**Figure 2:**
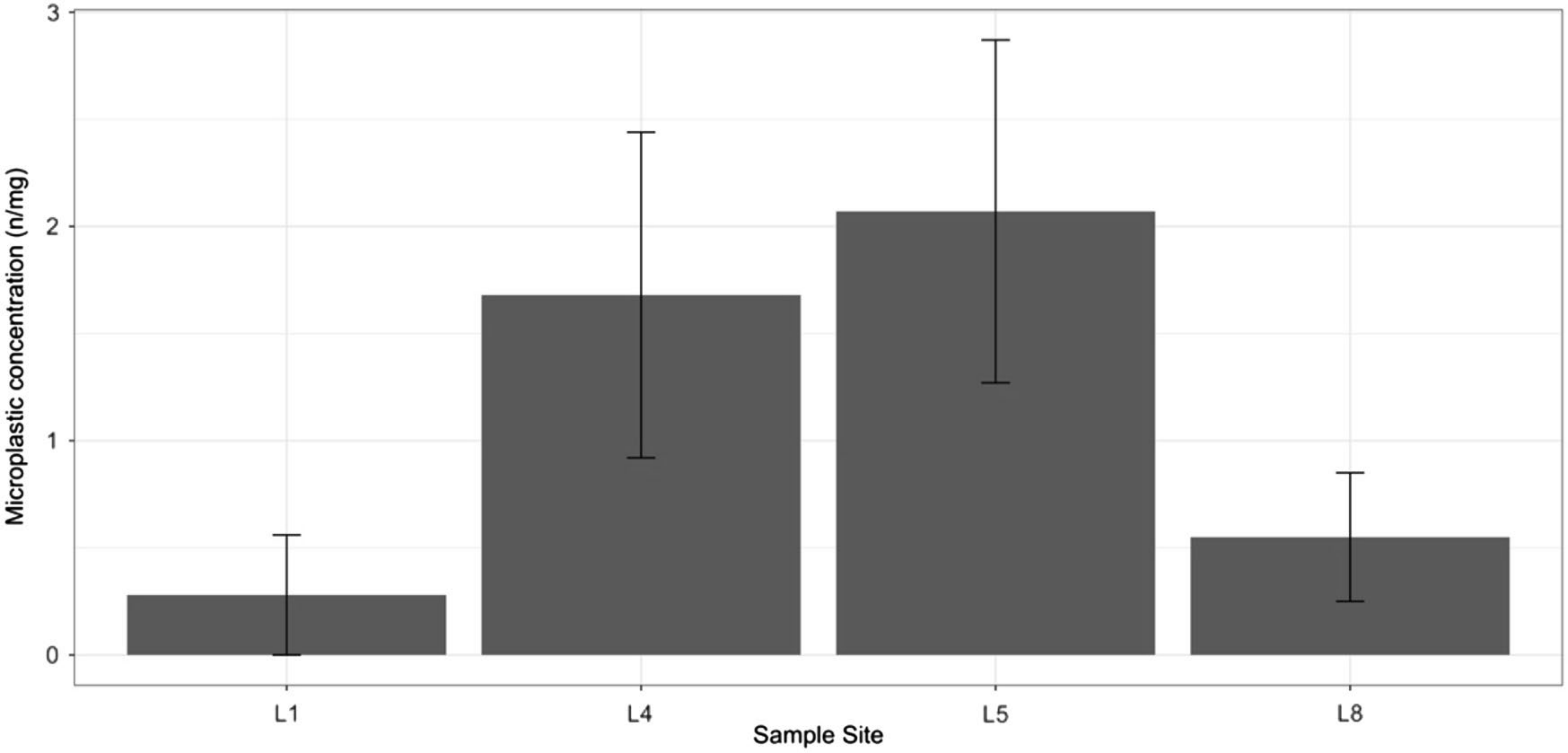
Mean microplastic concentration (n/mg) in Chironomidae larvae at sample sites (mean ± SD).

MP concentration in sample sites varied (Fig. 2). Site L0 was excluded in these comparisons due to no replication. MP concentration in the 4 sites were significantly different (one-way ANOVA, F_(3,8)_ = 6.454, *P* < 0.05); study sites L4 and L5 had significantly higher MP concentration compared to sites L1 and L8 (Post hoc LSD, *P* < 0.05). However, there were no significant differences between either sites L4 and L5, or sites L1 and L8. In the comparison of microplastic morphotypes, no significant differences between study sites were observed (Table S1; *P* > 0.05, microgranule: Kruskal–Wallis test, Chi-squared_(3)_ = 2.632; microfragment: Kruskal–Wallis test, Chi-squared_(3)_ = 2.961; microfilm: Kruskal–Wallis test, Chi-squared_(3)_ = 2.212; microfiber: one-way ANOVA, F_(3,8)_ = 1.87).

The relative abundance of 4 MP morphotypes varied at each sample site (Fig. 1). Microgranules accounted for 0–28% while they were not observed in site L5. Microfragments accounted for 3–47%. Microfilms accounted for 0–16%, while they were not observed in sites L4 and L8. Microfibers were the most common MP, accounting for 40–64%.

### Effect of anthropogenic activities

The industrial area influenced the total MP concentration in Chironomidae larvae. According to AICc, the proportion of industrial area (PIA) model was ranked as the best model (AICc: 39.3, *P* = 0.0185, beta = 18.8689), suggesting that PIA is a better variable for addressing our field data than the other variables. Positive beta supported the positive relationship between PIA and MP concentration. In contrast, AICc scores showed that the proportion of residential area (PRA) model did not give a description of the data that was as good as the PIA model (AICc: 41.9, *P* = 0.378, beta = 2.63). Additionally, we analyzed the relative importance (sum of weight) of variables by comparing models whose deltas were less than 4 (Table 2). The proportion and logarithm of industrial area are the most important variables (sum of weight: 0.32, 0.35, respectively), suggesting that industrial area influenced MP concentration in Chironomidae larvae greatly.

Residential area influenced the microfiber concentration in Chironomidae larvae. The results of the model selection highlighted that the proportion of residential area (PRA) model was the best model for description of microfiber concentration in midge larvae (AICc: 20.5, *P* < 0.001, beta = 22.314). Furthermore, the ratio of residential area to industrial area (RA/IA) model described the data of relative abundance of microfibers in sample sites with the least loss of information (AICc: -58.6, *P* = 0.156, beta = 0.103).

## Discussion

This study estimated the effects of different anthropogenic activities on the concentration of MPs in organisms in freshwater systems. Our results partially support the hypothesis as the industrial area was the most important variable, contributing the greatest to the MP concentration in Chironomidae larvae. In contrast, during the examination of the second hypothesis, despite no significant difference being found between microfiber concentration among sample sites, we found that the residential area mainly produced microfibers compared to other MP morphotypes. In addition, since we further suggested that dilution may occur for the relative abundance of microfibers, we examined the ratio of residential areas to industrial areas. The result suggests that the contribution of residential areas on the relative abundance of microfibers could be masked by industrial areas due to a greater production of MPs in the latter. Consequently, we proposed that industrial areas could be a potential source of MP pollution in river sediment, and Chironomidae larvae might be effective bioindicators in urban rivers due to their strong endurance in polluted water and the fact that they reflect MP concentration in river sediment with a broad size range of particles in their feeding habits.

According to Löder and Gerdts (2015), although we excluded transparent and white particles, visual identification may result in a high misidentification rate if it was applied to particles < 500 μm, which was mainly the size of particles in this study. However, all visual identification was conducted by the same person under same microscopy, thus, we assumed that misidentification rate was similar. Therefore, MP concentrations might be misestimated but consistent, and our major aim to assess the relationship between MP concentration and anthropogenic activity is still valid.

### Importance of industrial activities

By quantifying anthropogenic activities in an urban river catchment, we observed that industrial areas contributed a greater amount on the concentration of MPs in midge larvae than residential areas, as (1) the best mixed effect model consisted of the proportion of industrial areas in explaining the MP concentration, and (2) logarithm of the industrial area was the most important variable above all. We could observe this trend from the result that sites L4 and L5 have a high density of industrial area in the catchment (Table 1). In fact, industrial raw materials, including pellets, spherules, and flakes, were plastic debris which were commonly documented in the river system^46,47^. Lechner and Ramler (2015) claimed that 259.2 kg of industrial microplastics were legally discharged to river in Austria as the government considered microplastics as filterable material^48^. Therefore, it was not surprising that the industrial area made great contribution to MP concentration in the present study. However, we did not observe a high concentration of microgranules in the sample sites which included high industrial areas in the catchment. On the contrary, at site L5, the concentration of microgranule was 0 (Table S1). This was expected as it has been illegal to manufacture or import cosmetic products with microplastics since 2018 in Taiwan. The implication that abundant MPs from the industrial area might not be released from industry led to a discussion about the diffuse sources of MPs.

In order to enter the environment, the pathway of MPs can be categorized into point and diffuse sources^49^. Despite WWTPs being predicted as a potential source of MPs^14,50,51^, this study did not focus on point source (WWTPs). This is due to the face that WWTP effects on MP concentration could be simply insignificant^19^ or masked by diffuse sources that by-pass WWTPs^30^. These include plastics littering, combined sewer overflows (CSOs), and tire and road wear particles (TRWPs), which are all difficult to quantify^3^. Due to the impossibility of evaluating diffuse sources and ineffectiveness of evaluating point source, we determined that factors that can reflect MP inputs should not be confined with certain point source or behavior. Therefore, since human density is an equivocal indicator, a better indicator with a larger scale was necessary.

We suspected that diffused sources such as plastics littering, CSOs, and TRWPs may be possible inputs of MPs as we knew that a large amount of transportation (i.e. cement mixer trucks and dump trucks) and heavy traffic occurred daily in the industrial areas. In Siegfried et al. (2017), the model suggests that 42% of total MPs transported by rivers to sea were TRWPs, which highlights the necessity to collect complete information of daily traffic flow^52^. Besides, in Taiwan, CSOs were all connected to the river directly with the view of discharging storm water as soon as possible^53^, leading to large amount of MP inputs into the river system.

Since Chironomidae larvae reflected MP concentration in river sediment, the importance of industrial areas being a potential source of MP pollution in both surface water and sediments in urban areas is highlighted. Yonkos et al. (2014) investigated the correlation between MP on surface water and human activities, while the current study focused on organisms. Both studies observed similar results. Contrarily, Klein et al. (2015) found no increase of MP concentration in river sediment downstream of an industrial area. This contradictory observation might be explained by two things. First, instead of quantifying the industrial area of each sample site, Klein et al. (2015) simply pointed out the location of plastic processing in an industrial area and expected to observe elevated abundance of MP concentration downstream. Therefore, Klein et al. (2015) considered the industrial area as similar to a point source, which was contrary to our study. Second, the density of the industrial area was a better variable compared to the extent of the industrial area itself, suggesting that the catchment areas of each sample site were relevant and should be considered.

Addressing the research gap when studying MPs in the sediment system is essential. Although we have referenced previous research regarding MPs on surface water, other studies show that MP concentrations sampled from surface water are not able to support the hypothesis that MP level is associated with areas close to an anthropogenic source^54,55^. This might be explained by meteorological effects and hydrodynamics processes^56,57^. For instance, in Taihu Lake, China, Su et al. (2016) suggested that compared to surface water, MP concentration in sediment was more related to the distance from source. Another study at the surface of Rhine river ^55^ revealed that MP concentration in surface water in the urban area might decrease due to the enhancement of sedimentation rate resulting from slower flow velocity. Thus we believe that the knowledge of MPs in surface water limit our scientific perspectives, making it necessary to fill the research gap in river sediment. The heterogeneity of MP concentration in these two different sample circumstances influenced this study.

### Residential activities and microfibers

Although the concentration of microfibers did not differ significantly (Table S1), the model selection result suggested that the proportion of residential area (PRA) model was the best model to elucidate the concentration of microfibers in Chironomidae larvae (Table S2). This result supported Browne et al. (2011) who suggested that microfibers found in the marine environment are mainly discharged from washing machines, and washing machines are more closely associated with residential areas, not industrial areas. Thus, we were not surprised to observed the same pattern in Chironomidae larvae as MPs mostly enter the marine environment through rivers^19,20,58,59^. However, since we already observed the strong effect of industrial areas on total MP concentration in Chironomidae larvae, when we discussed the relative abundance of MP morphotypes at each sample site, it was very likely the contribution of microfibers on relative abundance of MP morphotypes would be masked by the industrial area. In fact, site L1, which was expected to show lower relative abundance of microfibers than site L5 due to a smaller residential area, showed a higher relative abundance of microfibers. Thus, before we conducted another mixed effect model selection on variables and relative abundance of microfibers, we created another variable, ratio of residential area to industrial area (RA/IA), representing the effect of residential area without dilution. The result supported our assumption as the RA/IA model was the best model to describe the relative abundance of microfibers in our data (Table S2). Overall, microfibers were popular and consisted of more than 40% of total MP in each sample site (Fig. 1), which was in accordance with previous field reports regarding freshwater species^24,25,54^. AICc scores suggested that residential areas influenced the degree of microfibers in Chironomidae larvae. This result supported our second hypothesis.

### Conclusion and overview

In conclusion, the major anthropogenic activities producing MPs that lead to urban river sediment pollution may be related to industrial areas according to our study. The future regulation of MP pollution should focus on industrial areas and an effective policy to lower the domestic MP pollution should include the mitigation of microfibers produced in residential area. This study proposes that (1) industrial areas are a potential source of MPs, and (2) Chironomidae are effective indicators of MPs in urban river systems as they are abundant even in polluted sites. In addition, we underline that the construction of complete land use data from the government should be constantly updated, allowing scientists to predict MP pollution in urban river systems.

Although there are no WWTPs that can eliminate MPs completely, they considerable reduce emissions of MPs in river systems^50,60^. As a result, the construction of more WWTPs in industrial parks and monitoring of MP pollution in rivers are short term solutions. In the long term, we need to improve the wastewater treatment methods to strengthen the last line of defense between MPs and the environment, and design CSO systems that can inhibit floods without carrying a high amount of MPs into the river system. The microfibers produced from washing machines are polluting deposit-feeders in urban river systems currently, leading to the need to assess detrimental effects of MPs on the development of riverine species (e.g., Chironomidae larvae) with environmental realism as soon as possible. Also, in order to quantify TRWPs, information of traffic flow history is necessary.

Future studies should be completed at larger scales to examine the accuracy and applicability of our hypotheses. Since agriculture creates considerable plastic debris in terrestrial areas, such as mulching, sludge usage, and seed film coating^61–63^, agricultural areas may be relevant for understanding MP concentration. In addition to a larger scale of sampling, characterization of WWTPs, better identification of MP particles, and seasonal differences in river flow, we need to discern what exactly leads to the high MP production in industrial areas. Moreover, different species ingest different amounts and sizes of MPs with various feeding strategies^27^. Despite the lack of comparisons in different Chironomidae species, such deposit-feeders might respond differently to MP particles because of their diverse ecological and biological traits^27,31,64^. Although this taxonomic difference did not influence our major results (i.e., the significant effects of anthropogenic activities), future efforts with this taxonomic consideration can provide better mechanistic understanding.

## Supplementary Information

Supplementary Information.
