# Supplementary material for: Effects of anthropogenic activities on microplastics in deposit-feeders (Diptera: Chironomidae) in an urban river of Taiwan
Source: Sci Rep. 2021 Jan 11;11:400. doi: 10.1038/s41598-020-79881-z (PMC7801685; doi:10.1038/s41598-020-79881-z)
Supplement: Supplementary file 1 — Supplementary Information. [file 41598_2020_79881_MOESM1_ESM.pdf]

Effects of anthropogenic activities on microplastics in deposit-feeders (Diptera: Chironomidae) in an urban river of Taiwan

Chun-Ting Lin[1], Ming-Chih Chiu[2*], Mei-Hwa Kuo[1*]

[1] Department of Entomology, National Chung Hsing University, Taiwan

[2] Institute of Hydrobiology, Chinese Academy of Sciences, China

* Corresponding author

Mei-Hwa Kuo: E-mail address: mhkuo@dragon.nchu.edu.tw

Ming-Chih Chiu: E-mail address: mingchih.chiu@gmail.com

Chun-Ting Lin: E-mail address: jordan841220@gmail.com

Supplementary data

Table S1. Comparison of Microplastic Concentration (n/mg) and Body Length (mm) of Chironomidae Larvae Between Sample Sites (Mean±SD).

Sample Sites	Concentration (n/mg)					Body length (mm)
	Microplastic	Microgranule	Microfragment	Microfilm	Microfiber	
**L0**	0.80	0.22	0.03	0.03	0.51	3.93 ± 0.93 c
**L1**	0.28 ± 0.28 b	0.14 ± 0.23 a	0.02 ± 0.03 a	0.003 ± 0.004 a	0.12 ± 0.05 a	4.35 ± 1.27 a
**L4**	1.68 ± 0.76 a	0.10 ± 0.17 a	0.45 ± 0.43 a	0.00 a	1.13 ± 1.19 a	3.83 ± 1.06 c
**L5**	2.07 ± 0.8 a	0.00 a	1.07 ± 1.07 a	0.21 ± 0.36	0.80 ± 0.23 a	3.52 ± 0.86 d
**L8**	0.55 ± 0.3 b	0.12 ± 0.21 a	0.24 ± 0.21 a	0.00 a	0.20 ± 0.21 a	3.79 ± 0.82 c
	One-way ANOVA	Kruskal-Wallis	Kruskal-Wallis	Kruskal-Wallis	One-way ANOVA	Kruskal-Wallis
	F(3,8) = 6.45	chi-squared(3) = 2.63	chi-squared(3) = 2.96	chi-squared(3) = 2.21	F(3,8) = 1.87	chi-squared(4) = 262.36
***p* value**	0.016	0.452	0.398	0.530	0.213	< 0.001

Numbers followed with different letters in same column indicate significant differences (One-way ANOVA, post hoc LSD test, $P<0.05$; Kruskal-Wallis test, post hoc Dunn's test, $P<0.05$). F(a,b) = F value with degree of freedom a and b. Chi-squared(a) = chi-squared with degree of freedom a.

Table S2. Generalized Linear Mixed Model: Relationship Between Variables and Relative Abundance of Microfibers and Microfiber Concentration (Random effects: sample dates).

**Fixed effects**	**Microfiber Concentration (n/mg)**								
	df	logLik	AICc	Delta	Conditional R^2	Marginal R^2	Beta	*p* value	Sum of weight
**PRA**	4	-3.40	20.5	0.00	0.7462	0.4251	22.3140	<0.001	0.47
**RA/IA**	4	-4.47	22.7	2.14	0.8267	0.4831	-0.5120	<0.001	0.16
**logIA**	4	-4.59	22.9	2.38	NA	0.2014	0.2605	0.022	0.14
**logRA**	4	-4.71	23.1	2.60	NA	0.1807	0.3153	0.016	0.13
**PIA**	4	-5.47	24.7	4.14	NA	0.1320	13.6090	0.118	0.06
**logCA**	4	-5.97	25.7	5.14	NA	0.0827	0.2769	0.177	0.04

**Fixed effects**	**Relative Abundance of Microfibers (%)**								
	df	logLik	AICc	Delta	Conditional R^2	Marginal R^2	Beta	*p* value	Sum of weight
**RA/IA**	4	23.30	-58.6	0.00	-0.6713	-0.5754	0.1034	0.156	0.63
**logIA**	4	21.23	-54.5	4.13	-0.6911	-0.0052	-0.0131	0.504	0.08
**logCA**	4	21.22	-54.4	4.14	-0.6866	-0.0015	0.0120	0.729	0.08
**PIA**	4	21.15	-54.3	4.29	-0.7119	-0.0179	-1.5243	0.294	0.07
**logRA**	4	21.07	-54.1	4.46	-0.6844	-0.0007	0.0064	0.814	0.07
**PRA**	4	21.04	-54.1	4.51	-0.6829	-0.0001	0.0675	NA	0.07

RA/IA: ratio of residential area to industrial area. logIA: logarithm of industrial area. logCA: logarithm of catchment area. PIA: proportion of industrial area. logRA: logarithm of residential area. PRA: proportion of residential area. df: degree of freedom. logLik: log likelihood. AICc: correction of AIC with small sample size. Conditional R^2: r-squared explained by both fixed factor(s) and random factors. Marginal R^2: r-squared explained by the fixed factor(s) alone. Sum of weights column indicates the relative importance of each variable.

Table S3. Generalized Linear Mixed Model: Relationship Between Variables and Microplastic Concentrations (Random effects: sample sites, sample dates).

Fixed effects	df	logLik	AICc	delta	weight	AIC	BIC	beta	*p* value	conditional R^2	marginal R^2
**PIA**	5	-10.35	39.3	0.00	0.34	30.70	33.52	18.8689	0.019	0.5882	0.4681
**logIA**	5	-10.46	39.5	0.23	0.31	30.93	33.75	0.2897	0.022	NA	0.5362
**logRA**	5	-11.11	40.8	1.53	0.16	32.23	35.05	0.3186	0.126	NA	0.4383
**logCA**	5	-11.60	41.8	2.50	0.10	33.20	36.03	0.2813	0.334	0.4998	0.1784
**PRA**	5	-11.67	41.9	2.63	0.09	33.33	36.15	5.6596	0.378	0.4603	0.1071
**logIA+logCA**	6	-8.17	42.3	3.06	0.06	28.33	31.72	logIA:0.7657<br>logCA:-0.8554	0.000<br>0.004	NA	0.6422
**PIA+PRA**	6	-9.47	44.9	5.66	0.02	30.93	34.32	PIA:17.0420<br>PRA:6.5230	0.006<br>0.139	0.6022	0.5752
**PRA+logIA**	6	-10.04	46.1	6.81	0.01	32.08	35.47	PRA:4.3313<br>logIA:0.2628	0.345<br>0.025	NA	0.5657
**PIA+logCA**	6	-10.27	46.5	7.27	0.01	32.54	35.93	PIA:22.1929<br>logCA:-0.1181	0.054<br>0.691	0.5873	0.4685
**PIA+logIA**	6	-10.28	46.5	7.28	0.01	32.55	35.94	PIA:11.8701<br>logIA:0.1198	0.537<br>0.691	0.5946	0.4895
**PIA+logRA**	6	-10.28	46.6	7.29	0.01	32.56	35.95	PIA:15.9020<br>logRA:0.0867	0.149<br>0.703	0.5950	0.4870
**logIA+logRA**	6	-10.34	46.7	7.41	0.01	32.68	36.07	logIA:0.4302<br>logRA:-0.2017	0.154<br>0.612	0.5879	0.4851
**logCA+logRA**	6	-10.80	47.6	8.33	0.01	33.61	37.00	logCA:-1.0323<br>logRA:1.1352	0.071<br>0.010	NA	0.6248
**PRA+logRA**	6	-11.00	48	8.73	0.00	34.01	37.40	PRA:3.2722<br>logRA:0.2788	0.730<br>0.214	0.5664	0.3754
**PRA+logCA**	6	-11.07	48.1	8.87	0.00	34.14	37.53	PRA:6.1868<br>logCA:0.2965	0.287<br>0.244	0.5499	0.3421
**logCA*logRA**	7	-7.46	51.3	12.05	0.00	28.92	32.87	logCA:-5.6973<br>logRA:-4.7798<br>logCA:logRA:0.3414	<0.001<br><0.001<br><0.001	NA	0.6641
**logCA*logIA**	7	-7.70	51.8	12.54	0.00	29.41	33.36	logCA:-0.0454<br>logIA:2.3322<br>logCA:logIA:-0.0819	0.883<br>< 0.001<br>0.000	NA	0.6583
**PIA*logIA**	7	-7.81	52.0	12.74	0.00	29.61	33.57	PIA:325.0195<br>logIA:0.0817<br>PIA:logIA:-18.3799	0.004<br>0.616<br>0.005	NA	0.6579
**logIA+logRA+logCA**	7	-7.89	52.2	12.92	0.00	29.79	33.74	logIA:0.6904<br>logCA:-1.0100<br>logRA:0.2155	0.001<br>0.005<br>0.459	NA	0.6523
**PIA*logCA**	7	-8.26	52.9	13.65	0.00	30.52	34.47	PIA:643.6820<br>logCA:0.1806<br>PIA:logCA:-33.0083	0.007<br>0.348<br>0.009	NA	0.6466
**PIA*PRA**	7	-8.46	53.3	14.04	0.00	30.91	34.87	PIA:-63.3680<br>PRA:-18.4215<br>PIA:PRA:1250.1346	0.284<br>0.316<br>0.174	NA	0.6432
**PIA*logRA**	7	-8.62	53.6	14.36	0.00	31.23	35.19	PIA:891.4989<br>logRA:0.4673<br>PIA:logRA:-54.0505	0.017<br>0.017<br>0.019	NA	0.6371
**PRA*logIA**	7	-8.87	54.1	14.86	0.00	31.73	35.69	PRA:-68.3935<br>logIA:-0.1490<br>PRA:logIA:5.9910	0.098<br>0.491<br>0.067	0.6646	0.6112
**PIA+PRA+logCA**	7	-9.22	54.8	15.56	0.00	32.43	36.39	PIA:20.6540<br>PRA:8.1353<br>logCA:-0.1535	0.005<br>0.031<br>0.441	NA	0.6172
**PRA*logCA**	7	-9.27	54.9	15.67	0.00	32.54	36.50	PRA:-129.5095<br>logCA:-0.2084<br>PRA:logCA:8.3762	0.045<br>0.403<br>0.029	0.6769	0.5889
**PIA+PRA+logRA**	7	-9.27	54.9	15.67	0.00	32.54	36.50	PIA:20.3640<br>PRA:9.6273<br>logRA:-0.1262	0.007<br>0.058<br>0.490	NA	0.6146
**PRA*logRA**	7	-9.32	55.0	15.76	0.00	32.63	36.59	PRA:-132.6889<br>logRA:-0.1742<br>PRA:logRA:9.5066	0.050<br>0.445<br>0.037	NA	0.6463
**PIA+PRA+logIA**	7	-9.46	55.3	16.04	0.00	32.91	36.87	PIA:18.7553<br>PRA:6.9940<br>logIA:-0.0325	0.222<br>0.264<br>0.904	NA	0.5924
**PIA+PRA+logIA+logRA**	8	-7.18	66.4	27.08	0.00	30.35	34.87	PIA:-38.8747<br>PRA:13.6127<br>logIA:1.8975<br>logRA:-1.6161	0.125<br>0.001<br>0.015<br>0.011	NA	0.6762
**PIA+PRA+logIA+logCA**	8	-7.18	66.4	27.08	0.00	30.35	34.87	PIA:-30.2386<br>PRA: -4.0265<br>logIA:1.5812<br>logCA:-1.5070	0.172<br>0.448<br>0.016<br>0.011	NA	0.6762

PIA: proportion of industrial area. logIA: logarithm of industrial area. logRA: logarithm of residential area. logCA: logarithm of catchment area. PRA: proportion of residential area. df: degree of freedom. logLik: log likelihood. AICc: correction of AIC with small sample size. BIC: Bayesian Information

Criterion. Conditional R^2: r-squared explained by both fixed factor(s) and random factors. Marginal R^2: r-squared explained by the fixed factor(s) alone. Sum of weights column indicates the relative importance of each variable.

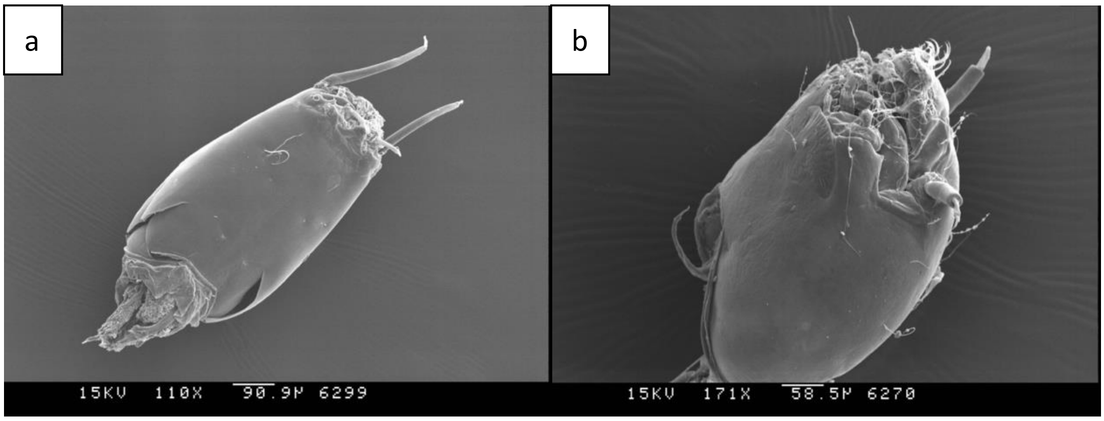

Fig S1. Details of larval head capsule: ventral aspect of (a) *Thienemannimyia* spp. and (b) *Chironomus* spp.

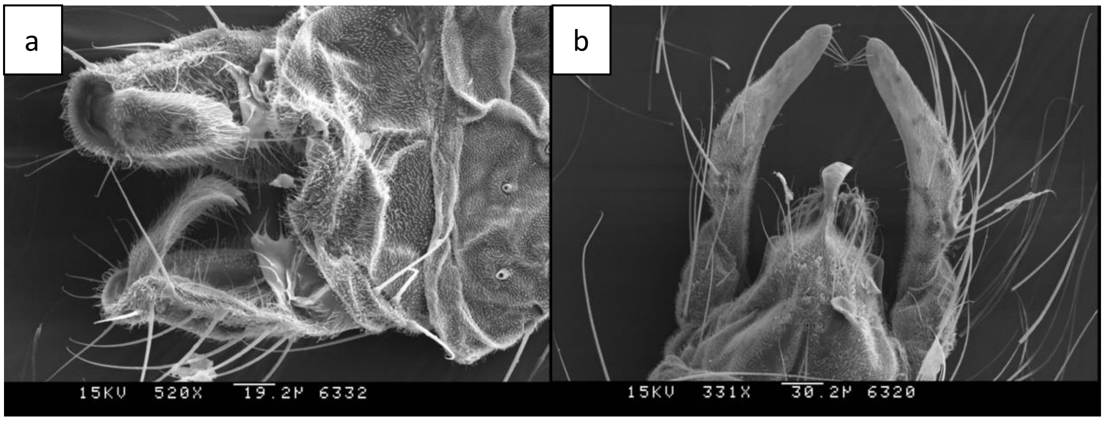

Fig S2. Terminalia of male: (a) *Orthocladius* spp. and (b) *Chironomus* spp.

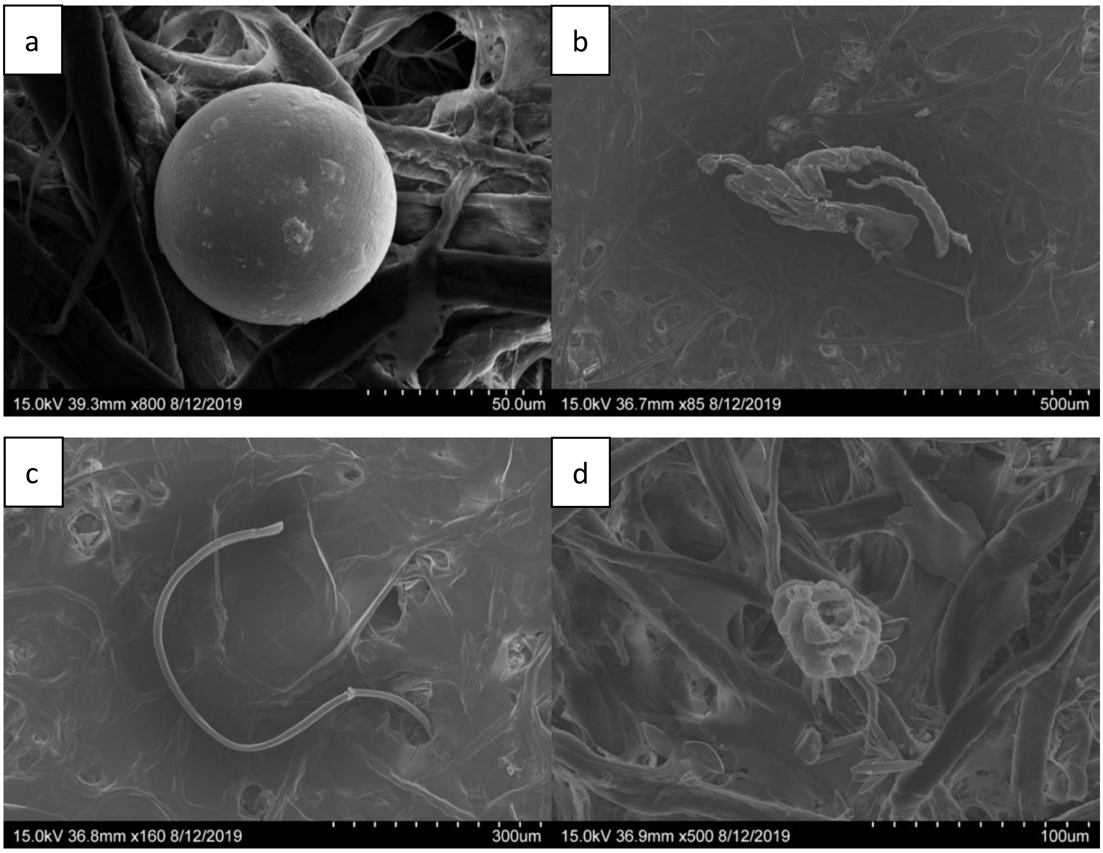

Fig. S3. An array of microplastics by 4 categories of shape. (a) microgranule; (b) microfilm; (c) microfiber; (d) microfragment.
